# Who is protected and who is not? Evaluating the effects of the long-term care insurance system and disparities in healthcare utilization and costs of the South Korean elderly population

**DOI:** 10.1186/s12913-025-12747-0

**Published:** 2025-07-01

**Authors:** Minsung Sohn, Hojoon Sohn, Mankyu Choi

**Affiliations:** 1 Division of Health and Medical Sciences, The Cyber University of Korea, Seoul, South Korea; 2 Department of Preventive Medicine, Seoul National University College of Medicine, Seoul, South Korea; 3 Institute of Health Policy and Management, Seoul National University, Seoul, South Korea; 4 Department of Human Systems Medicine, Seoul National University College of Medicine, Seoul, South Korea; 5 Department of Health Policy & Management, College of Health Science, Korea University, Seoul, South Korea

**Keywords:** Long-term care insurance (LTCI), Long-term care service (LTCS), Health care utilization, Medical costs, Income disparities, Older adults, Policy evaluation, Difference-in-differences analysis

## Abstract

**Background:**

There is a growing global interest in investigating the substitutability between long-term care service (LTCS) and medical care use, particularly in countries where elderly medical care use is rapidly increasing. The use of an appropriate LTCS can reduce unnecessary medical utilization and the resulting costs such as those for hospital admission, delayed discharge, and emergency room visits.

**Methods:**

This study evaluated whether the LTCS has a savings effect on medical use for older adults and whether the effect varies depending on income level, using longitudinal data obtained from an Elderly Cohort Database compiled from 2002 to 2013, which is a sample of 10% of the elderly population in Korea. This study also included 70,437 older adults who participated during the 2002–2007 (pre-policy) and 2009–2013 (post-policy) periods.

**Results:**

The results show that the use of inpatient and long-term care hospitals decreased significantly in LTCS users compared with non-users; additionally, the length of stay and medical costs were lower. However, since the introduction of the system, the use of outpatient and acute care hospitals (ACH) by LTCS users has increased slightly compared with non-users. Low-income LTCS users experienced more cost-sharing in outpatient and ACH than high-income users.

**Conclusions:**

Although LTCI system could reduce the hospitalization rate for the older adults using LTCS, more attention to middle- and low-income populations is necessary as they may be within the blind spot of security.

## Introduction

Population aging is rapidly progressing worldwide. An aging population can pose considerable challenges in the provision of health and social care, including concerns about a rapid increase in healthcare services among the geriatric population, thereby imposing considerable stress on healthcare systems and financing of the National Health Insurance (NHI) Scheme. South Korea is no exception to this growing global trend and tops the countries in the Economic Co-operation and Development (OECD) regarding geriatric population growth and costs [1]. Subsequently, the share of medical care costs for the older adults has been increasing over the years; by 2021, this estimate had reached 43.4% (KRW 40.6 trillion, US$ 354.6 hundred million) of the total medical costs reported by the National Health Insurance Service [2].

For the elderly population, effective linkage and delivery of medical treatment and elderly care are critical in preventing the worsening of existing complications or the development of emergency medical situations, which can cause considerable financial stress on both health systems and patients [3, 4]. In 2008, the South Korean government established long-term care insurance (LTCI) to address the rapidly growing concerns of the aging population and the increasing gap between medical and elderly care services. By formalizing reimbursements for what was once an informal care service for older adults, the South Korean LTCI covers healthcare and elderly care services provided through both institutional and home-based care, including those offered by family members [5].

There is a growing interest in investigating the relationship between long-term care service (LTCS)/LTCI and medical care use in countries with rapidly increasing elderly medical care use [6]. Studies have shown that increases in LTCSs can reduce medical service utilization and associated costs [7, 8], particularly by reducing hospital stays [9], unnecessary hospitalization [10], and emergency room visits [11]. Home-based end-of-life care can also be highly cost-saving [12–14]. However, these effects may not be generalizable to lower socioeconomic/income populations, who are more likely to experience social admission and may be at a greater risk of experiencing disparities in the financial burden associated with health service use [15]. Increases in benefits coverage for health insurance tend to benefit higher socioeconomic tier populations [16, 17].

Hence, our study aimed to address several limitations of earlier South Korean research that evaluated the association between LTCI/LTCS and medical utilization [18, 19]. These limitations include the cross-sectional study design, difficulties in evaluating long-term effects, and lack of assessment of the effects of the LTCI/LTCS on medical care utilization based on income level. Therefore, we conducted a longitudinal study using data from 2002 to 2013 to evaluate the performance of the South Korean LTCI regarding modifications in medical service use and the associated medical care cost-savings for older adults. Specifically, we examined the effect of LTCI on medical care utilization, such as length of stay (LOS) and total medical costs, for LTCS users compared with non-users after implementing LTCI from 2002 to 2013, stratified by income level.

## Materials and methods

### Study design and data preparation

The South Korean LTCI offers various benefits, such as support for daily living and physical activities, to older adults, particularly for those suffering from geriatric diseases. The demand for LTCI has been steadily rising, with 9.47% of the elderly population using it by 2021, which increased from 6.5% in 2013 [20]. The LTCI involves a selective application process to determine eligibility for grades ranging from 1 to 5, dementia cognitive support grades, and eligibility for facility or in-home care. Elderly people with severe physical or mental disabilities, grades 1 and 2, will primarily benefit from facility-based care. Applicants for LTCI are evaluated for their care recognition score based on physical function, cognitive function, etc., and those who score below a certain standard are disqualified from being selected as LTCS users. As of 2021, approximately 74.4% of the applicants were granted LTCI [21].

LTCI’s main purpose is to cover services for the prevention of illness or post-treatment care provided in facilities or at home, whereas the NHI primarily covers medical services to treat illnesses [22]. Despite these efforts to distinguish between the types of services covered by each program, many individuals continue to use various healthcare services without distinguishing between treatment and care. Additionally, the costs of medical care and LTCI for older adults have increased, contradicting the role of LTCI in reducing medical expenses.

We utilized the NHIS-Senior Cohort (NHIS-SC) database, an administrative database consisting of 10% random sampling of individuals aged > 60 years since 2002, totaling 558,147 older adults. Our study used data established in 2013, including LTCI data from July 2008. We merged the NHIS-SC and LTCI data using personal identifiers such as the national registration number. The dataset excluded individuals without records of LTCS use or LTCI application. The dataset included information on subscriber qualifications, periodic health examination records, sociodemographic and economic status, chronic and other disease status, and medical care usage and costs [20].

### Participants

**Fig. 1:**
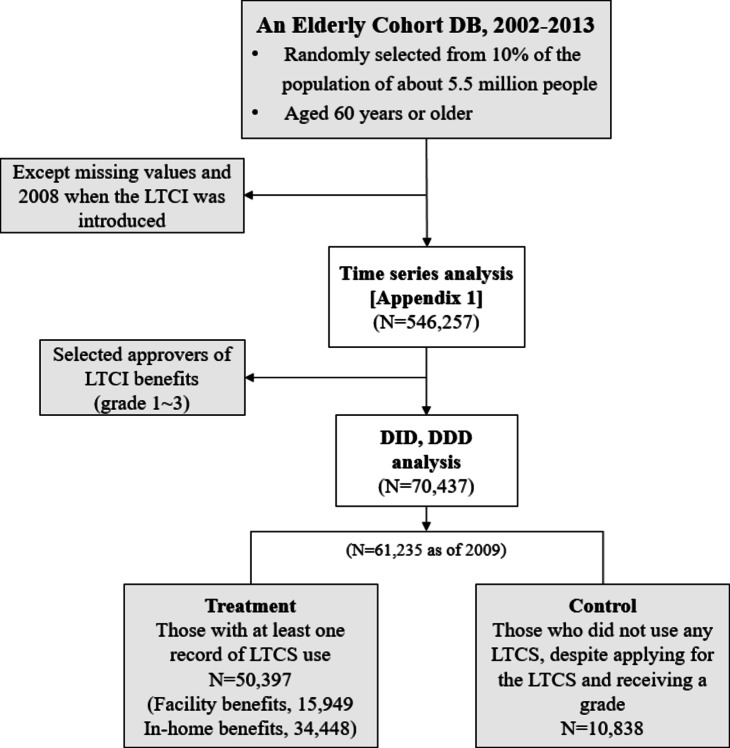
Flowchart of sample selection

An overview of the participant selection process is presented in Fig. 1. From the original NHIS-SC and LTCI merged databases for 2002 through 2013, we excluded 11,661 people because of missing values or lack of responses regarding the main variables. To clarify the effect of LTCI, we considered those during 2002–2007 as the pre-policy period and those during 2009–2013 as the post-policy period—except for 2008, when LTCI was introduced (*N* = 546,257). The final dataset included data from 70,437 older adults before performing additional analyses. Among the elderly who applied for LTCI, only those who were approved for LTCS (grade 1 to 3) were selected, and a total of 70,437 people were selected for the difference-in-differences (DID) and difference-in-difference-in-differences (DDD) analyses. Those who used an LTCS at least once were classified into the treatment group, and those who had never used an LTCS were classified into the control group. For example, as of 2009, of 61,235 patients, there were 50,397 and 10,838 in the treatment and control groups, respectively (Fig. 1).

### Key variables

The main independent variables for our study were assigned to individuals based on their exposure to the LTCI policy, categorized as pre-policy(0) or post-policy [1], and policy benefit status, categorized as non-user (0) or user [1]. LTCI users were defined by long-term care grade, ranging from 1 to 3, as determined by the Long-Term Care Grading Committee, and included those with records of LTCS use. The control group consisted of non-users who applied for LTCI but had no records of LTCS use despite receiving a grade. Age was categorized into six groups in five-year increments. Furthermore, we classified individuals’ residences into two major categories: major metropolitan cities (Busan, Daegu, Daejeon, Gyeonggi, Incheon, Kwangju, Seoul, and Ulsan) and small cities or rural areas (Chungnam, Chungbuk, Gyeongbuk, Gyeongnam, Kangwon, Jeju, Jeonnam, Jeonbuk, and Sejong). We utilized the income deciles used to determine individual and household NHI contribution levels as a proxy for income status and categorized this information into five different income levels: high income (top 10%), middle high income (10–30%), middle low income (30–70%), low income (bottom 30%), and medical aid beneficiaries. An individual’s disability status was categorized as those with any disability (“yes”) or those without disability (“no”). The level of comorbidities was assessed based on the Charlson comorbidity index (CCI), which represents the sum of a weighted index of 1–6 points of 17 disease groups that considers the number and seriousness of preexisting comorbid conditions [23]. In Korea, medical cost structure is regulated under the NHI system, where the government sets standardized medical fees for insured services. The medical fees are adjusted upward on an annual basis to account for factors such as inflation, increased operational costs, and advancements in medical technology. When analyzing patterns of medical use, it is essential to control for the effect of rising medical fees as a variable. Therefore, we used the number of medical use and rate of change in medical fee that could affect the health care utilization in the analysis of policy effect (Appendix 1).

We applied the International Classification of Diseases- 10 codes to classify health services use and costs by types of disease the individuals sought medical care for. The total costs for each type of healthcare service and prescription drug consumed were assessed by multiplying the frequency of service use by service unit costs, as reported in the NHI fee-for-service reimbursement schedule, which is renewed each year. We assessed the individuals’ cumulative healthcare service costs as the sum of all total healthcare service costs calculated within the timeframe of our analysis. LOS and total medical cost data were log-transformed to account for nonnormal (skewed) distributions. For LOS and medical costs data with no observed value, we added a value of “1” to these observations to allow for log-transformation (e.g., log(LOS + 1)).

### Statistical analyses

The main outcomes considered in this study were LOS and total medical costs, which have also been considered in previous research evaluating the effects of LTCI/LTCS [7, 24]. We used these dual outcome measures to evaluate the impact of the LTCI/LTCS from the perspective of health systems. LOS was applied to gauge the degree of medical service use; total medical costs, adjusted for the frequency of use, reflected the strength and quality of medical use. Healthcare service utilization was further categorized as inpatient/outpatient and acute care or long-term care hospitals (LTCH) based on the types of medical services and institutions providing medical services. Data on LTCS types and service use were categorized based on the location of the service provision: facilities or in-home benefits. Facility benefits included any services provided at the long-term care (LTC) facilities. The in-home benefits include home-visit care, home-visit bathing, home-visit nursing, day and night care, and short-term care, except for welfare equipment services. Users of both facility and in-home services in subsequent years, accounting for approximately 2% of the total, were excluded from the analysis.

### DID and DDD analysis

This study aimed to evaluate the impact of the LTCI policy on healthcare utilization among South Korean older adults. We utilized the DID analysis method, which is commonly used to assess policy effects in various fields, including healthcare policy [25, 26]. DID analysis compare outcome measures between an intervention group and a control group, either before and after policy implementation or between two different periods. The main output of the DID analysis was the net policy effect obtained by subtracting the natural effect of the control group from that of the intervention group.

First, we verified the parallel trend assumption before conducting the DID analysis. Additionally, a time series analysis was conducted to examine the trends in LOS and total medical costs for the overall elderly population and LTCI approvers (Appendix 2). We applied DID analysis to examine the effects of the LTCI policy on the quarterly length of hospital stay and total medical costs per older adult. We compared the pre- and post-policy changes among the three groups of LTCS users (total service users, facility benefit users, and in-home benefit users) and a control group of non-users. We also categorized the sample into five groups based on healthcare utilization patterns: total users, inpatient care users, outpatient care users, ACH users, and LTCH users. The net policy effect was estimated by calculating the pre-post differences in our outcome measures based on the following model equation:

$$\begin{aligned}\text{Y}_{(i,t)}&={\upbeta}_0+{\upbeta}_1{(\text{post})}_{t}+{\upbeta}_2{(\text{treatment})}_{i}\\&+{\upbeta}_3{(\text{post x treatment})}_{{it}}\\&+{\upbeta}_4{(\text{individual confounders})}_{{it}}+\upvarepsilon_{{it}}\end{aligned}$$

*i*: individual, *t*: quarter of the year, 

 Y: LOS, total medical costs,

 individual confounders: sex, age, residence, health insurance type, disability, the number of CCI, medical fee, and the number of medical use

To assess the effect of the policy on different income levels, we utilized the DDD method and expanded the DID analysis to include an additional control group. Income level (determined by the household’s health insurance premium) and health insurance types [27] were included as control variables by adding interaction terms to the DID model. We also added other predisposing determinants such as sex, age, and residence to the model. This allowed us to estimate the differences in the net policy effects of the LTCI on income levels in the elderly population [27]:

$$\begin{aligned}\text{Y}_{(i,t)}&={\upbeta}_0+{\upbeta}_1{(\text{post})}_{t}+{\upbeta}_2{(\text{treatment})}_{i}+{\upbeta}_3{(\text{income level})}_{{it}}\\&+{\upbeta}_4{(\text{post x treatment})}_{{it}}+{\upbeta}_5{(\text{post x income level})}_{it}\\&+{\upbeta}_6{(\text{treatment x income level})}_{{it}}+{\upbeta}_7{(\text{post x treatment x income level})}_{it}\\&+{\upbeta}_8{(\text{individual confounders})}_{{it}}+\upvarepsilon_{{it}}\end{aligned}$$

*i*: individual, *t*: quarter of the year,

Y: LOS, cost-sharing,

individual confounders: sex, age, residence, health insurance type, disability, the number of CCI, medical fee, and the number of medical use

## Results

### Characteristics of samples

We used cohort data for two 11-year periods from 2002 to 2007 (pre-policy) and 2009 to 2013 (post-policy), excluding 2008, to assess the long-term effects of LTCI since its introduction in this year. The final sample of 70,437 older adults approved for LTCI were included in our analysis. Table 1 presents the study cohort characteristics used to analyze the effect of the policy on healthcare utilization in the 2009 (*N* = 61,235), considered the post-policy baseline period. We further classified the population into three groups: LTCI non-users, those with facility benefits, and those with in-home benefits.

Table 1 provides the general cross-sectional characteristics of the elderly population included in the final 2009 dataset. Of the 61,235 elderly users of LTCS in 2009, 10,838 were deemed non-users, 15,949 utilized facility benefits at least once, and 34,448 were considered in-home benefit users. The sex distribution in this cohort year was similar to that observed in 2002. Those aged > 80 years were still the most frequent users of facility services. The majority of the elderly population in all three groups resided in the metropolitan area, and there were slight differences among non-users, facility benefit users, and in-home benefit users at 61.82%, 60.23%, and 64.15%, respectively. The medical aid group had the largest share (24.47%) among the facility users. Older adults with disabilities were distributed in the following order: facilities (3.56%), in-home (3.36%), and non-users (2.51%), and the number of CCI was similar for the three groups. The number of medical uses was highest in the in-home benefit group, followed by LTCS non-users and the facility benefit group. Those with the most severe LTCS grade (grade 1) were 33.32% in the facility benefit group but were a relatively minor group for the non-user (22.14%) and in-home benefit (14.67%) groups.

**Table 1:** Characteristics of the samples (Policy effect analysis, *N* = 61,235 (2009))

	Non-user (*N* = 10,838)		Facility benefits (*N* = 15,949)		In-home benefits (*N* = 34,448)	
	*N*	(%)	*N*	(%)	*N*	(%)
Sex						
Men	3,752	(34.62)	3,651	(22.89)	10,724	(31.13)
Women	7,086	(65.38)	12,298	(77.11)	23,724	(68.87)
Age						
65–69	848	(7.82)	922	(5.78)	3,060	(8.88)
70–74	2,180	(20.11)	2,587	(16.22)	7,423	(21.55)
75–79	2,725	(25.14)	3,846	(24.11)	9,050	(26.27)
80–84	2,602	(24.01)	4,114	(25.79)	7,883	(22.88)
85 and over	2,483	(22.91)	4,480	(28.09)	7,032	(20.41)
Residence						
Large city	6,700	(61.82)	9,606	(60.23)	22,098	(64.15)
Small city or rural	4,138	(38.18)	6,343	(39.77)	12,350	(35.85)
Income level						
High-income	2,148	(19.82)	2,516	(15.78)	7,152	(20.76)
Middle high-income	2,643	(24.39)	3,308	(20.74)	8,232	(23.90)
Middle low-income	2,635	(24.31)	3,424	(21.47)	7,921	(22.99)
Low-income	1,967	(18.15)	2,799	(17.55)	6,029	(17.50)
Medical aid	1,445	(13.33)	3,903	(24.47)	5,114	(14.85)
Disability						
No	10,566	(97.49)	15,381	(96.44)	33,292	(96.64)
Yes	272	(2.51)	568	(3.56)	1,156	(3.36)
^a^The number of CCI	2.44(1.08)	0–13	2.40(1.78)	0–11	2.47(1.83)	0–12
^a^The number of medical use	7.58(8.15)	1–89	6.63(7.96)	1–104	8.64(9.82)	1–133
LTCI Grade						
Grade 1	2,399	(22.14)	5,314	(33.32)	5,052	(14.67)
Grade 2	2,656	(24.51)	6,050	(37.93)	7,120	(20.67)
Grade 3	5,783	(53.36)	4,585	(28.75)	22,276	(64.67)

^a^ mean, SD, range

### The impact of the LTCI on the LOS and total medical costs

Table 2 presents the DID estimation of the impact of LTCS on hospital LOS and the total medical costs for older adults. Adjusted DID estimate via addition of the interaction term “post-policy x service user” after controlling for covariates showed that the LOS and total medical costs of older adults using LTCS were 29% and 24% lower, respectively, compared to non-LTCS users before the policy introduction.

According to the type of hospital and service, the LOS and medical costs of LTCH users increased by 16% and 21%, respectively, after the introduction of the LTCI; however, adjustment using the interaction term demonstrated that an older adult who used LTCS after the introduction of the system had reduced LOS and medical costs relative to the levels observed prior to its introduction. In the case of outpatient and acute care uses, those who used in-home benefits had longer LOS and higher medical costs than LTCS non-users. When considering the interaction term in our model, post-policy implementation LTCS use was associated with reductions in the LOS and medical expenses (LOS: outpatient β=− 0.18, acute care β=− 0.20; total medical costs: outpatient β=− 0.05, acute care β=− 0.08) compared to the non-users (prior to the policy implementation).

**Table 2:** The impact of the LTCI on the LOS and total medical costs of beneficiaries compared with non-user: DID estimation results

		**LOS**						**Total medical costs**					
		**Total user**		**Facility benefits**		**In-home benefits**		**Total user**		**Facility benefits**		**In-home benefits**	
		^**a**^ **β**	**SE**	^**a**^ **β**	**SE**	^**a**^ **β**	**SE**	^**a**^ **β**	**SE**	^**a**^ **β**	**SE**	^**a**^ **β**	**SE**
**Total**	**After** (Ref: Before)	-.11^***^	.002	-.10^***^	.002	-.11^***^	.002	-.09^***^	.002	-.08^***^	.003	-.08^***^	.003
	**Use** (Ref: Non-user)	-.17^***^	.004	-.04^***^	.003	-.16^***^	.004	-.03^***^	.003	-.25^***^	.007	-.11^***^	.006
	**After** **x** **Use** (Ref: Before x Non-user)	-.29^***^	.004	-.35^***^	.008	-.28^***^	.004	-.24^***^	.006	-.35^***^	.008	-.20^***^	.006
	Adjusted R square	37.3%		33.9%		38.2%		35.0%		33.9%		35.8%	
**Inpatient care**	**After** (Ref: Before)	-.01	.006	.01	.007	.01	.006	.01	.006	.02^*^	.007	.01	.007
	**Use** (Ref: Non-user)	-.15^***^	.006	-.10^***^	.007	-.19^***^	.006	-.09^***^	.006	-.13	.007	-.07^***^	.006
	**After** **x** **Use** (Ref: Before x Non-user)	-.11^***^	.008	-.08^***^	.010	-.15^***^	.009	-.06^***^	.009	-.11^***^	.010	-.05^***^	.009
	Adjusted R square	47.7%		49.9%		48.2%		39.4%		40.9%		39.8%	
**Outpatient care**	**After** (Ref: Before)	-.19^**^	.001	-.19^**^	.001	-.20^**^	.001	-.17^***^	.002	-.17^***^	.002	-.18^***^	.002
	**Use** (Ref: Non-user)	.01^**^	.003	-.01	.001	.02^**^	.003	.09^***^	.005	.01	.006	.12^***^	.006
	**After** **x** **Use** (Ref: Before x Non-user)	-.18^***^	.003	-.19^***^	.004	-.18^***^	.003	-.08^***^	.006	-.15^***^	.007	-.05^***^	.006
	Adjusted R square	56.2%		56.2%		56.7%		38.7%		38.5%		39.5%	
**Acute care**	**After** (Ref: Before)	-.17^***^	.001	-.17^***^	.002	-.17^***^	.002	-.14^**^	.002	-.15^**^	.003	-.13^**^	.002
	**Use** (Ref: Non-user)	-.03^***^	.003	-.05^***^	.004	-.06^***^	.003	.02^**^	.005	-.06^**^	.007	.05^**^	.006
	**After** **x** **Use** (Ref: Before x Non-user)	-.20^***^	.003	-.22^***^	.004	-.20^***^	.004	-.12^***^	.006	-.20^***^	.007	-.08^***^	.006
	Adjusted R square	47.6%		46.9%		48.7%		38.2%		37.6%		39.1%	
**Long-term care**	**After** (Ref: Before)	.16^***^	.011	.15^***^	.013	.27^***^	.013	.21^***^	.015	.20^***^	.017	.34^***^	.017
	**Use** (Ref: Non-user)	-.75^***^	.015	-.96^***^	.017	-.59^***^	.015	-.94^***^	.020	-1.25^***^	.024	-.72^***^	.021
	**After** **x** **Use** (Ref: Before x Non-user)	-.20^***^	.021	-.35^***^	.023	-.25^***^	.021	-.20^***^	.028	-.41^***^	.031	-.26^***^	.029
	Adjusted R square	18.3%		23.1%		24.7%		18.6%		23.3%		25.8%	

^a^β means adjusted sex, age, residence, income level, health insurance type, disability, CCI, medical fee, and number of medical use

^*^p<0.05, ^**^p<0.01, ^***^p<0.001

### The impact of LTCI on cost-sharing by income levels

The effect of LTCS implementation on health service utilization and cost was largest in the lowest-income groups, as demonstrated by the large reduction in cost sharing in this group relative to the highest-income group (Table 3). In particular, this effect was mostly driven by the large decrease in cost sharing for hospitalization and the use of long-term care facility services in the lowest income group. Contrastingly, the effect of LTCS implementation on the decrease in cost sharing was smaller for outpatient and acute care service use.

**Table 3:** The impact of LTCI on cost-sharing by income levels compared with non-users: DDD Estimation results

	Total		Inpatient care		Outpatient care		Acute care		Long-term care	
	_a_β	SE	_a_β	SE	_a_β	SE	_a_β	SE	_a_β	SE
**Policy x Service x Income** (Ref: After x User x High-income)	1		1		1		1		1	
Middle high-income	− 0.042^***^	0.008	− 0.084^***^	0.016	− 0.030^***^	0.007	− 0.031^***^	0.007	− 0.052^***^	0.030
Middle low-income	− 0.124^***^	0.008	− 0.140^***^	0.017	0.013^***^	0.007	− 0.106^***^	0.008	− 0.168^***^	0.031
Low-income	− 0.309^***^	0.008	− 0.414^***^	0.018	0.166^***^	0.007	0.059^***^	0.008	− 0.447^***^	0.033
Medical aid	− 2.508^***^	0.010	− 12.095^***^	0.027	− 1.518^***^	0.009	− 2.011^***^	0.010	− 7.603^***^	0.039
Adjusted R square	44.0%		84.4%		45.2%		44.1%		65.0%	

_a_β means adjusted sex, age, residence, income level, health insurance type, disability, CCI, medical fee, and number of medical use

^*^p < 0.05, ^**^p < 0.01, ^***^p < 0.001

## Discussion and implications

This study evaluates the long-term performance and effectiveness of the LTCI policy on changes in medical service use over an eight-year period following the introduction of the national program. This study determines that LTCS use is associated with a reduced LOS and medical costs for inpatient or long-term care users compared to non-users. However, the study also reveals a slight increase in outpatient and acute care service utilization for LTCS users following the implementation of the system. Our study further highlights a large gap in LTCS use among the elderly population in lower-income groups, despite the government implementing LTCI policies to expand benefit coverage and improve universal healthcare coverage.

### LTCI policy effect on medical care utilization

After the introduction of the LTCI program, LOS and total medical costs decreased by 29% and 24%, respectively, for inpatient care and LTCH users compared to non-users. It is noteworthy that although the LTCS is not designed to replace hospital-based care, it can provide an essential mechanism for reducing unnecessary hospital stays, thereby reducing the overall medical costs for inpatient care [19, 28]. Our findings suggest that, despite the availability of LTCS, non-LTCS users who did not utilize LTCS instead accessed medical services at LTCH. This indicates that if the elderly had appropriately used LTCS, unnecessary medical utilization could have been reduced [7, 9, 18].

Meanwhile, since the introduction of the LTCI system, LTCS users have slightly increased their use of medical services compared to non-users, particularly in home care services. These are consistent with the findings of Ricauda et al. [28, 29] who reported that outpatient costs increased after the introduction of LTCI for the older adults. These research findings highlight the importance of monitoring the medical utilization and health status of elderly individuals receiving LTC in-home benefits. In the future, it will be necessary to establish an efficient linkage between LTC in-home benefits and medical care.

Additionally, this study shows that older adults using facility services experience a greater cost-saving effect than those using in-home services in both inpatient and outpatient care use, as well as in both ACH and LTCH. In other words, it was confirmed that elderly individuals utilizing LTC facility services were particularly effective as an alternative to long-term hospitalization. This suggests that the appropriate use of LTCS contributes to the efficient allocation of medical resources by reducing instances of ‘social hospitalization’. Unlike facility benefits, the use of in-home benefits does not serve as a substitute for LTCH, which could lead to an increase in the LOS and medical costs [30–32]. Therefore, there is a necessity for the proper management of human resources, such as clinical and ethics education for home-based service providers. In conclusion, this study provides evidence that the introduction of the national LTCI program has led to a reduction in medical costs and LOS for inpatient or long-term care users compared to non-users.

### LTCI policy effect on medical care utilization by income level

Our study established that, first, the decrease in cost sharing for those receiving medical aid was much larger than that for the high-income group, despite experiencing longer hospital stays and higher total medical costs [33, 34]. This may be explained by the fact that medical aid beneficiaries receive additional socioeconomic protective services through the LTCI and the NHI system relative to other health insurance subscribers. Second, implementation of the LTCI policy led to a decrease in cost-sharing with hospitals for LTCS users compared to service non-users, but the effect of protecting older adults from cost-sharing was more significant for low-income LTCS users than for high-income users. Cost-sharing in outpatient and ACH for LTCS users decreased across all income groups, but the reduction was more pronounced among low-income LTCS users compared to their high-income counterparts. Relatively, this policy did not effectively reduce the cost-sharing gap between income levels for outpatient and ACH services compared to inpatient care and LTCH. Similarly, previous studies have shown that the extension of NHI benefit coverage does not improve income-related equality in healthcare utilization [15, 35]. This suggests that the substitution effect of medical services using LTCS was not sufficiently realized, especially among low-income elderly individuals, excluding those in the medical aid group. The smaller reduction in cost-sharing among the low income group may be affected by their economic status. The proportion of health care expenditures to income would increase to a greater extent at the low-income level even if health care utilization or medical costs increased at high-income as much as low-income level. As this indicates, an LTCI system with a standard cost-sharing rate may not provide adequate protection for low-income elderly against financial burden. That is, elderly people with a low-income level have trouble using LTCS due to the burden of costs even if they are LTC beneficiaries, which leads to unmet need for medical care. Even if the LTC service is used, a vicious cycle may be repeated in which the amount of medical service use in acute care, where treatment is inevitable, is increased. Therefore, it is necessary to consider both the level of income and the beneficiaries in terms of equity of medical burden in the operation of this system.

## Conclusion

Our study findings have significant implications for interpreting the impact and gaps in the LTCI policy on medical care utilization, which vary according to the type of medical service, LTCS, hospitals, and income levels. We observed that the South Korean LTCI policy led to reductions in LOS and medical costs for inpatient care and/or LTCH. However, we also noticed that policy impact gaps existed by income level, assessed in terms of cost-sharing between LTCS users and non-users. This is a crucial concern, as those in lower-income groups may face barriers to accessing both LTCS and medical care services. Additionally, for countries considering implementing and expanding LTCS coverage, the long-term sustainability of the LTCI system should be carefully considered.

## Figures and Tables

**Figure:**
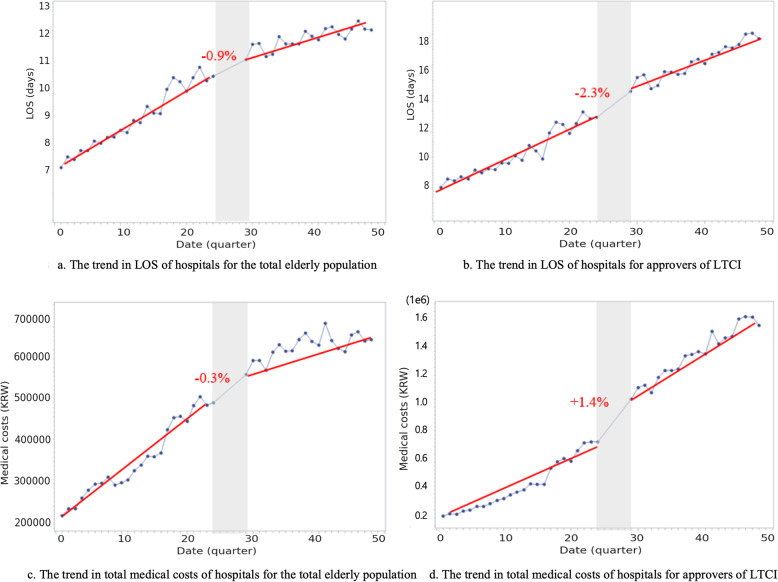
The trend in LOS and total medical costs of hospitals for the total elderly population or approvers of LTCI

## Data Availability

The dataset analyzed for this study is only available when permitted upon application due to the National Health Insurance Corporation’s data regulations related to the concerns of confidentiality for study subjects. The data supporting the findings of this study are available from “NHIS-Senior Cohort (NHIS-SC) database (2002 to 2013)” for the longitudinal study of aging by the National Health Insurance Service at https://nhiss.nhis.or.kr/.

## Appendix 1

**Table:** Medical Fee Index, 2002-2013

**Year**	**2002**	**2003**	**2004**	**2005**	**2006**	**2007**	**2008**	**2009**	**2010**	**2011**	**2012**	**2013**
Rate of increase (%)	0	2.97	2.65	2.99	3.58	2.30	1.94	2.22	2.05	1.64	2.20	2.36
Reflection of medical fee	100.00	102.97	105.62	108.61	112.19	114.49	116.43	118.65	120.70	122.34	124.54	126.90

Source: 2014 National Health Insurance Statistics (NHIS, 2015)

## Abbreviations

ACH: Acute care hospitals
CCI: Charlson comorbidity index
DDD: Difference-in-difference-in-differences
DID: Difference-in-differences
LOS: Length of stay
LTC: Long-term care
LTCH: Long-term care hospitals
LTCI: Long-term care insurance
LTCS: Long-term care service
NHI: National Health Insurance
NHIS: National Health Insurance Service
NHIS-SC: NHIS-Senior Cohort

## References

[1] Organisation for Economic Co-operation and Development (OECD). OECD Health Statistics 2022. OECD; 2022. Available from: https://www.oecd.org/health/health-data.htm.

[2] National Health Insurance Service (NHIS). 2021 Korean Health Insurance Statistics. NHIS; 2022. Available from: https://www.nhis.or.kr.

[3] van Oostrom SH, Picavet HS, de Bruin SR, Stirbu I, Korevaar JC, Schellevis FG, et al. Multimorbidity of chronic diseases and health care utilization in general practice. BMC Fam Pract. 2014;15:61. 24708798 10.1186/1471-2296-15-61 PMC4021063

[4] Gonzalez-Jaramillo V, Fuhrer V, Gonzalez-Jaramillo N, Kopp-Heim D, Eychmüller S, Maessen M. Impact of home-based palliative care on health care costs and hospital use: A systematic review. Palliat Support Care. 2021;19(4):474–87. 33295269 10.1017/S1478951520001315

[5] Kim H, Kwon S. A decade of public long-term care insurance in South Korea: policy lessons for aging countries. Health Policy. 2021;125(1):22–6. 33189411 10.1016/j.healthpol.2020.11.003

[6] World Health Organization (WHO). World report on ageing and health. Geneva: WHO; 2015. Available from: https://www.who.int/publications/i/item/9789241565042.

[7] Forder J. Long-term care and hospital utilisation by older people: an analysis of substitution rates. Health Econ. 2009;18(11):1322–38. 19206085 10.1002/hec.1438

[8] Maetens A, Beernaert K, De Schreye R, Faes K, Annemans L, Pardon K, et al. Impact of palliative home care support on the quality and costs of care at the end of life: a population-level matched cohort study. BMJ Open. 2019;9(1):e025180. 30670524 10.1136/bmjopen-2018-025180 PMC6347879

[9] Lichtenberg FR. Is home health care a substitute for hospital care? Home Health Care Serv Q. 2012;31(1):84–109. 22424308 10.1080/01621424.2011.644497

[10] Johri M, Beland F, Bergman H. International experiments in integrated care for the elderly: a synthesis of the evidence. Int J Geriatr Psychiatry. 2003;18(3):222–35. 12642892 10.1002/gps.819

[11] Smith ER, Stevens AB. Predictors of discharges to a nursing home in a hospital-based cohort. J Am Med Dir Assoc. 2009;10(9):623–9. 19883884 10.1016/j.jamda.2009.06.003

[12] Chiang JK, Kao YH. Impact of home hospice care on patients with advanced lung cancer: A longitudinal Population-Based study in Taiwan. J Palliat Med. 2016;19(4):380–6. 26618516 10.1089/jpm.2015.0278

[13] Ferroni E, Avossa F, Figoli F, Cancian M, De Chirico C, Pinato E, et al. Intensity of integrated primary and specialist Home-Based palliative care for chronic diseases in Northeast Italy and its impact on End-of-Life hospital access. J Palliat Med. 2016;19(12):1260–6. 27697009 10.1089/jpm.2016.0158

[14] Lustbader D, Mudra M, Romano C, Lukoski E, Chang A, Mittelberger J, et al. The impact of a Home-Based palliative care program in an accountable care organization. J Palliat Med. 2017;20(1):23–8. 27574868 10.1089/jpm.2016.0265 PMC5178024

[15] Kim S, Kwon S. The effect of extension of benefit coverage for cancer patients on health care utilization across different income groups in South Korea. Int J Health Care Finance Econ. 2014;14(2):161–77. 24691773 10.1007/s10754-014-9144-y

[16] Aron-Dine A, Einav L, Finkelstein A, Cullen M. MORAL HAZARD IN HEALTH INSURANCE: DO DYNAMIC INCENTIVES MATTER? Rev Econ Stat. 2015;97(4):725–41. 26769985 10.1162/REST_a_00518 PMC4710379

[17] Kim JH, Lee KS, Yoo KB, Park EC. The differences in health care utilization between medical aid and health insurance: a longitudinal study using propensity score matching. PLoS ONE. 2015;10(3):e0119939. 25816234 10.1371/journal.pone.0119939 PMC4376904

[18] Kim MKS, Kim H. The effect of long-term care utilization on health care utilization of the elderly. Korean J Health Econmics Policy. 2013;19:1–22.

[19] Jung WSYE. The effect on health care utilization of the Non-Use of beneficiaries of Long-Term care insurance Service - around of geriatric hospital’s medical cost. J Korea Academia-Industrial Cooperation Soc. 2015;16:7463–73.

[20] National Health Insurance Service (NHIS), Big Data Operation Office. Older Adult Cohort DB 2.0 User Manual (ver 1.0). NHIS; 2022. Available from: https://nhiss.nhis.or.kr.

[21] National Health Insurance Service (NHIS), Long-Term Care Insurance (LTCI). 2021 Statistical Yearbook of National Health Insurance and Long-Term Care Insurance. NHIS; 2022. Available from: https://www.nhis.or.kr

[22] Shin H. Comparison between the Aged Care Facilities Provided by the Long-Term Care Insurance (LTCI) and the Nursing Hospitals of the National Health Insurance (NHI) for Elderly Care in South Korea. Healthcare (Basel). 2022;10(5):779. 10.3390/healthcare10050779 PMC9140364 35627917

[23] Hwang SM, Yoon SJ, Ahn HS, An HG, Kim SH, Kyeong MH, et al. [Usefulness of comorbidity indices in operative gastric cancer cases]. J Prev Med Public Health. 2009;42(1):49–58. 19229125 10.3961/jpmph.2009.42.1.49

[24] Hutchinson AF, Parikh S, Tacey M, Harvey PA, Lim WK. A longitudinal cohort study evaluating the impact of a geriatrician-led residential care outreach service on acute healthcare utilisation. Age Ageing. 2015;44(3):365–70. 25536957 10.1093/ageing/afu196

[25] Chen L, Yip W, Chang MC, Lin HS, Lee SD, Chiu YL, et al. The effects of Taiwan’s National health insurance on access and health status of the elderly. Health Econ. 2007;16(3):223–42. 16929478 10.1002/hec.1160

[26] Polsky D, Doshi JA, Escarce J, Manning W, Paddock SM, Cen L, et al. The health effects of medicare for the near-elderly uninsured. Health Serv Res. 2009;44(3):926–45. 19674430 10.1111/j.1475-6773.2009.00964.x PMC2699915

[27] L Y. Factors of long term care service use by the elderly. Health Social Welf Rev. 2009;29:213–35.

[28] Lee HMY. The effect of Long-term care utilization on health care utilization of the elderly the Korean. J Health Econ Policy. 2015;21:81–102.

[29] Aimonino Ricauda N, Tibaldi V, Leff B, Scarafiotti C, Marinello R, Zanocchi M, et al. Substitutive hospital at home versus inpatient care for elderly patients with exacerbations of chronic obstructive pulmonary disease: a prospective randomized, controlled trial. J Am Geriatr Soc. 2008;56(3):493–500. 18179503 10.1111/j.1532-5415.2007.01562.x

[30] Condelius A, Hallberg IR, Jakobsson U. Medical healthcare utilization as related to long-term care at home or in special accommodation. Arch Gerontol Geriatr. 2010;51(3):250–6. 20006391 10.1016/j.archger.2009.11.009

[31] Dobrzyn-Matusiak D, Marcisz C, Bąk E, Kulik H, Marcisz E. Physical and mental health aspects of elderly in social care in Poland. Clin Interv Aging. 2014;9:1793–802. 25364237 10.2147/CIA.S69741 PMC4211863

[32] Wysocki A, Kane RL, Dowd B, Golberstein E, Lum T, Shippee T. Hospitalization of elderly medicaid long-term care users who transition from nursing homes. J Am Geriatr Soc. 2014;62(1):71–8. 24383662 10.1111/jgs.12614

[33] Sohn M, Jung M, Choi M. Self-Rated health status based on the type of health insurance: A socioeconomic perspective. Inquiry. 2021;58:469580211028171. 34218705 10.1177/00469580211028171 PMC8261851

[34] Sohn M, Jung M. Effects of public and private health insurance on medical service utilization in the National health insurance system: National panel study in the Republic of Korea. BMC Health Serv Res. 2016;16(1):503. 27654146 10.1186/s12913-016-1746-2 PMC5031343

[35] Kim S, Kwon S. Has the National health insurance improved the inequality in the use of tertiary-care hospitals in Korea? Health Policy. 2014;118(3):377–85. 25459734 10.1016/j.healthpol.2014.10.005

